# An Uncommon Affection in a Limb, from an Apparent Slight Accident

**Published:** 1803-03-01

**Authors:** James Lucas

**Affiliations:** Conisbrough, Member of the College of Surgeons in London


*An uncommon* Affection *in a* Limb, *from an apparent slight Accident; by Mr.* James Lucas, *of Conisbrough, Member of the College of Surgeons in London.*

Miss F. in the twenty-third year of her age, after having walked in snow, and neglected to change her dress, was seized with fever and sore throat succeeded not long after by pains in the arms and knees. Before these complaints were removed, and being in a weak state, she had the misfortune, from her foot having been entangled in her cloaths, to fall forwards upon the chamber floor. She became unable to reach the bed without help, and passed a very restless night. The next morning she had so much pain in the hip, that is was necessary to ascertain whether any material injury had ensued. The limb admitted of every natural motion, and I was perfectly convinced that there was neither dislocation nor fracture. In ten days the pain and stiffness being considerably abated, she attempted to walk, and observed that the weakness of the hip joint excited a dread of falling.

Electricity, embrocations, frictions, riding on horseback, and internally bark and steel, were diligently tried, yet in two or three weeks, there being scarcely any perceptible amendment as to her lameness, she became very much dispirited, and particularly on being convinced of the following circumstances by her own experiments.

"When she walked she was obliged to describe a half circle, as those do who wear artificial legs that are made longer than they ought to be; in standing as strait as she could, the lame hip was higher, and the knee turned outward; in a sitting posture, with the limbs placed upon a sopha, by a tape measure the sound limb was unquestionably shorter, yet, by pressing the foot on the lame side against the sopha, she could make the length of the limbs exactly alike."

At the head of the femur, and of the great trochanter, instead of the natural prominence, there was also an unusual hollowness.

Being at that time a young practitioner, I so far sympathized with the anxious apprehension of my patient, as secretly to blame myself for not having been more inquisitive as to the progress of this malady. On mature deliberation I began to be more emboldened, predicting that these signs might be owing to a relaxation of the ligament, allowing the head of the femur to slip down upon the inner and lower edge of the acetabulum; that the effects of such an accident might be more violent, from the habit having been disposed to rheumatism, and having been weakened by indisposition; that, considering her youth, there was every reason to hope for eventual success.

The first examination had been satisfactory, the motions of the limb had, throughout the complaint, and at present, afforded strong proofs that there had never existed either fracture or luxation.

She became by degrees more confident in the expectation of a cure; and when she began to be sensibly better, she could in ten days walk with ease, all the formidable symptoms gradually vanished, and in a week or two more she was perfectly well.

The most sudden and effectual relief appeared to be received by a warm plaister, which eventually proved vesicatory, (Emp. commun. ℥j. pulv. euphorb. emp. cantharid. aa. ʒj. m.)

In the course of near thirty years practice, I do not recollect to have met with more than one somewhat similar case; where a gentleman, of nearly the same age, during rheumatism after a typhus fever, had a slip, but did not fall, which was succeeded by a protraction, and other like appearances, with a lameness, which was not so tedious, and seemed to admit of remedy by local exercise of the limb, in the manner recommended by Buzzalio for gouty patients. When I was at Bath, I saw an electrical machine, well calculated for weak limbs being exercised and electrified at the same time.

The favourable termination of such cases should encourage professors of the art not to relax in their endeavours to invent probable remedies, lest invalids should be driven to have mischief done by the more violent measures of ignorant pretenders.

EXPERIMENTS

